# Comprehensive diffusion MRI dataset for in vivo human brain microstructure mapping using 300 mT/m gradients

**DOI:** 10.1038/s41597-021-01092-6

**Published:** 2022-01-18

**Authors:** Qiyuan Tian, Qiuyun Fan, Thomas Witzel, Maya N. Polackal, Ned A. Ohringer, Chanon Ngamsombat, Andrew W. Russo, Natalya Machado, Kristina Brewer, Fuyixue Wang, Kawin Setsompop, Jonathan R. Polimeni, Boris Keil, Lawrence L. Wald, Bruce R. Rosen, Eric C. Klawiter, Aapo Nummenmaa, Susie Y. Huang

**Affiliations:** 1 Athinoula A. Martinos Center for Biomedical Imaging, Department of Radiology, Massachusetts General Hospital, Charlestown, Massachusetts United States; 2 Harvard Medical School, Boston, Massachusetts United States; 3 Department of Neurology, Massachusetts General Hospital, Boston, Massachusetts United States; 4 Harvard-MIT Division of Health Sciences and Technology, Massachusetts Institute of Technology, Cambridge, Massachusetts United States; 5 Department of Life Science Engineering, Institute of Medical Physics and Radiation Protection, Giessen, Germany

**Keywords:** Brain imaging, Cognitive ageing, Magnetic resonance imaging

## Abstract

Strong gradient systems can improve the signal-to-noise ratio of diffusion MRI measurements and enable a wider range of acquisition parameters that are beneficial for microstructural imaging. We present a comprehensive diffusion MRI dataset of 26 healthy participants acquired on the MGH-USC 3 T Connectome scanner equipped with 300 mT/m maximum gradient strength and a custom-built 64-channel head coil. For each participant, the one-hour long acquisition systematically sampled the accessible diffusion measurement space, including two diffusion times (19 and 49 ms), eight gradient strengths linearly spaced between 30 mT/m and 290 mT/m for each diffusion time, and 32 or 64 uniformly distributed directions. The diffusion MRI data were preprocessed to correct for gradient nonlinearity, eddy currents, and susceptibility induced distortions. In addition, scan/rescan data from a subset of seven individuals were also acquired and provided. The MGH Connectome Diffusion Microstructure Dataset (CDMD) may serve as a test bed for the development of new data analysis methods, such as fiber orientation estimation, tractography and microstructural modelling.

## Abstract (summary)

**Table:** 

Measurement(s)	brain measurement
Technology Type(s)	diffusion magnetic resonance imaging
Factor Type(s)	diffusion time • gradient strength • direction
Sample Characteristic - Organism	Homo sapiens

Machine-accessible metadata file describing the reported data: 10.6084/m9.figshare.16910290

## Background & Summary

Diffusion magnetic resonance imaging (MRI) offers a unique and valuable tool for mapping brain tissue microstructure and structural connectivity noninvasively in the living human brain. Diffusion MRI benefits from strong gradient systems that achieve more efficient diffusion-encoding^1–3^, resulting in decreased echo time and higher signal-to-noise ratio (SNR), and also enabling a larger diffusion measurement space to be probed *in vivo*. As part of the Human Connectome Project (HCP), the MGH-USC Consortium engineered the first 3 T whole-body Connectome MRI scanner equipped with 300 mT/m maximum gradient system – the strongest gradients ever built for a human MRI scanner^1,2^. Such high gradient strengths have benefited diffusion tractography by enhancing the resolution of complex fiber configurations for improved reconstruction of white matter fiber pathways^4–9^. An important by-product of such dedicated high gradient systems is the unparalleled assessment of brain tissue microstructure *in vivo* through sensitizing the diffusion MRI signal to water diffusion within highly restricted compartments, thereby enabling the mapping of microstructural properties such as axon diameter, density and g-ratio^7,10–22^. In recognition of the importance of high gradient strengths for *in vivo* tissue microstructural mapping, an ultra-strong gradient human MRI scanner with even higher maximum amplitude of 500 mT/m is currently being developed as part of the NIH BRAIN Initiative-funded Connectome 2.0 Project^23,24^.

Despite the advantages of strong gradient systems for human imaging, data acquisition on such systems requires specialized expertise and resources, rendering the data less accessible. Currently, only four Connectome MRI scanners have been installed worldwide^1–3^. As part of the efforts of the HCP, we previously released the MGH–USC Adult Diffusion Dataset from 35 healthy adult participants^8^, which consisted of four *b*-values up to 10,000 s/mm^2^ acquired at a single diffusion time with relatively dense angular sampling that was mainly designed for mapping the human structural connectome^25–30^. In this work, we leverage the high gradient strengths and wide range of diffusion times accessible on the Connectome scanner to acquire the MGH Connectome Diffusion Microstructure Dataset (CDMD) consisting of data from 26 healthy adult participants, which samples a wider range of *b*-values up to ~18,000 s/mm^2^ and multiple diffusion times. This comprehensive dataset will benefit the development of new analytic frameworks and models for mapping human brain tissue microstructure, as well as serving as a normative high-gradient strength dataset for exploring changes in brain tissue microstructure and structural connectivity across the lifespan.

The acquisition protocol was designed to sample the available diffusion measurement space. Specifically, the hour-long acquisition included two diffusion times (19 and 49 ms), eight shells corresponding to gradient strengths linearly spaced between 30 mT/m and 290 mT/m per diffusion time, and maximum *b*-value of 17,800 s/mm^2^. The acquisition used a custom-built 64-channel phased-array head coil to boost image SNR^31^, cutting-edge in-plane acceleration incorporating a Fast Low angle Excitation Echo Train to suppress parallel imaging artifacts due to breathing and head motion during the auto-calibration signal acquisition^32^, simultaneous multi-slice (SMS) for accelerated whole-brain coverage^33–37^, and an expert diffusion-weighted pulse sequence enabling independent control of different sequence parameters, and reconstruction of magnitude and phase images. Moreover, scan/rescan data in a subset of seven healthy individuals were acquired to assess the variability in diffusion MRI estimates extracted from this protocol^38^.

Portions of the dataset have been used to develop new methods for mapping axon diameter and density^17,19,38^ and to investigate alterations in axonal microstructure in multiple sclerosis^10,39^ and the aging brain^12^, as well as explore the diffusion time dependence of estimated microstructural metrics and fiber orientations^24,40^. This high-quality dataset may be used as a test bed for the development of new fiber orientation estimation, tractography and microstructural modelling methods, including methods for probing gray matter microstructure such as soma and neurite density imaging (SANDI)^41^ that may benefit from data acquired with high gradient strengths, which are often developed and initially tested in small animals on preclinical scanners. The MGH CDMD thus provides an important opportunity for the translation of such methods to human imaging. The two diffusion times sampled in the acquired diffusion dataset may be employed to explore diffusion time dependence in different tissues^42–46^ and the effects of inter-compartmental water exchange in gray matter^47–49^. Finally, atlases of microstructural metrics derived from high-gradient diffusion MRI can be created using the group data.

## Methods

### Participants

The institutional review board of Mass General Brigham approved the study protocol and ensured that all relevant ethical regulations were complied with. Written informed consent was obtained from all participants. Data were acquired in 26 healthy, cognitively normal adults (22 to 72 years old, 36.8 ± 14.6 years old, 17 females and 9 males). Seven out of 26 participants (22 to 72 years old, 37.1 ± 18.4 years old, 4 females) were scanned twice in the same day. For the scan/rescan experiments, participants were removed from the scanner after the first scan session and were given a rest period of approximately one hour^38^. They were then repositioned within the scanner for the second scan session.

### Data acquisition

All data were acquired on the 3 T Connectome MRI scanner (Magnetom CONNECTOM, Siemens Healthineers) equipped with a maximum gradient strength of 300 mT/m and maximum slew rate of 200 mT/m/ms. The slew rate was de-rated to a maximum of 62.5 mT/m/ms during diffusion encoding to prevent peripheral nerve stimulation. A custom-built 64-channel phased array head coil^31^ was used for signal reception.

Diffusion MRI data were acquired using a two-dimensional single-refocused diffusion-weighed pulsed-gradient spin-echo echo planar imaging (EPI) sequence^50,51^ with anterior-to- posterior phase-encoding direction. For each participant, 66–70 contiguous sagittal slices were acquired with the center slice placed along the midline corpus callosum to achieve symmetric whole brain coverage. The imaging parameters are as follows: repetition time (TR) = 3800 ms, echo time (TE) = 77 ms, field of view (FOV) = 216 × 216 mm, matrix size = 108 × 108, slice thickness = 2 mm, voxel size = 2 × 2 × 2 mm^3^, diffusion time (Δ) = 19 or 49 ms, diffusion-encoding gradient duration (δ) = 8 ms, eight diffusion-encoding gradient strengths evenly spaced between 30 and 290 mT/m (i.e., 31, 68, 105, 142, 179, 216, 253, 290 mT/m) for each diffusion time corresponding to 16 different b-values (i.e., 50, 350, 800, 1500, 2400, 3450, 4750, and 6000 s/mm^2^ for Δ = 19 ms; 200, 950, 2300, 4250, 6750, 9850, 13,500, 17,800 s/mm^2^ for Δ = 49 ms), 32 diffusion encoding directions uniformly distributed on a sphere for b < 2400 s/mm^2^ and 64 uniform directions for b ≥ 2400 s/mm^2^ respectively, one interspersed b = 0 image volume for every 16 diffusion-weighted images (DWIs) (i.e., 50 b = 0 images and 800 DWIs in total), parallel imaging using generalized autocalibrating partially parallel acquisitions (GRAPPA)^32,52^ with an acceleration factor R = 2, simultaneous multislice^33,35–37^ (SMS) acceleration factor = 2, partial Fourier factor = 7/8, bandwidth = 2315 Hz/pixel. The choice of δ = 8 ms was based on limitations to the accessible rise time imposed by the scanner hardware and the need for sufficiently long delta to achieve high b-values > 10,000 s/mm^2^. The 32 and 64 diffusion-encoding directions were identical for each diffusion time and each gradient strength. Five b = 0 image volumes with reversed phase-encoding direction (posterior-to-anterior) were also acquired to correct for susceptibility-induced image distortions. The acquisition time for the entire diffusion MRI protocol was 55 minutes.

In 14 participants (a total of 21 scan or rescan sessions), an adapted version of the same diffusion MRI sequence was used for acquisition to enable the export of the phase images in addition to the magnitude images. Real-valued diffusion data were derived by removing the nuisance background phase (reflecting contributions from physiological noise, motion, and instrumental imperfections such as inhomogeneity in the B_1_-transmit field) from the raw complex image data and then taking the real part following previous studies^19,53–55^.

T_1_-weighted structural MRI data were acquired using a 3-dimensional multi-echo magnetization-prepared gradient echo (MEMPRAGE) sequence^56^ with whole-brain coverage. The imaging parameters are as follows: TR = 2530 ms, TE = 1.15, 3.03, 4.89, 6.75 ms, inversion time (TI) = 1100 ms, flip angle = 7°, 176 contiguous sagittal images, FOV = 220 × 220 mm, matrix size = 220 × 220 mm, 1 mm isotropic resolution, GRAPPA R = 3, bandwidth = 650 Hz/pixel, acquisition time = 4 minutes.

### Data processing

**Fig. 1:**
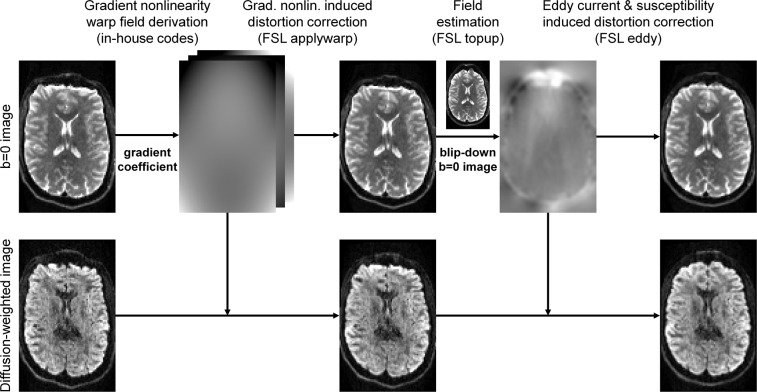
Diffusion data processing pipeline. Diffusion data were corrected for gradient nonlinearity, susceptibility and eddy current induced image distortions and co-registered.

The diffusion data processing pipeline is outlined in Fig. 1. The diffusion data were first corrected for gradient nonlinearity-induced image distortions using in-house Matlab codes^8^ and the proprietary gradient coefficients provided by Siemens. The corrected images were computed using the derived warp fields and the “applywarp” function from the FMRIB Software Library (FSL)^57^ with the cubic spline interpolation.

The diffusion data were then corrected for susceptibility and eddy current-induced image distortions and co-registered using the TOPUP^58^ and EDDY^59,60^ functions (i.e., the GPU version “eddy_cuda9.1”) from FSL (version v6.0.4). The TOPUP function was performed with default parameters and the pre-defined configuration file “b02b0.cnf” provided by FSL. The pre-defined configuration file was slightly modified to adjust the spatial resolution of the warp field for the last two iterations from 4 mm isotropic to 2 mm isotropic, the native spatial resolution of the diffusion MRI data. Specifically, the line “--warpres = 20,16,14,12,10,6,4,4,4” in the “b02b0.cnf” file was modified to “--warpres = 20,16,14,12,10,6,4,2,2”. The EDDY function was performed with dynamic susceptibility correction^61^, slice-to-volume motion correction^62^ and default parameters except for the cubic eddy current model, eight iterations, and considering both positive and negative outliers (i.e., “--ol_pos option” option). Since the EDDY function treats data as separate shells only when their b-values differ by more than 100 s/mm^2^, the acquired b-values of 50, 200 and 2400 s/mm^2^ were slightly modified to 101, 202 and 2401 s/mm^2^ respectively to enforce differentiation between shells with b-values of 0, 101 and 2300 s/mm^2^, such that the data from each acquired shell could be separately modelled and corrected for eddy current effects. The modified b-values were only used for the purpose of utilizing the EDDY function properly in the step of data pre-processing and the b-values released in the data sharing repository are true to the acquisition. The brain mask used for the EDDY function was obtained by binarizing the sum of all brain masks created from each interspersed b = 0 image volume using FSL’s “bet2”^63^ function with the fractional intensity threshold set to 0.1. The modified diffusion-encoding directions from EDDY (i.e., provided by the “my_eddy_output.rotated_bvecs” output file) were used to account for the head motion of participants, resulting in slightly varying diffusion-encoding directions for each participant.

For the slice-to-volume motion correction in the EDDY function, the “--mporder” was set to the number of excitations in a volume (i.e., number of image slices in a volume over 2 due to the SMS acceleration factor equal to 2) over 2 and the “--s2v_lambda” was set to 1. The EDDY function was designed to be more sensitive to outliers at lower b-values (i.e., 50, 200, 350 s/mm^2^ in this work) and was sometimes too sensitive to find any image volumes without outlier slices as the shape reference volume for the slice-to-volume motion correction. To circumvent algorithm abortions, the image volumes at b-values of 50, 200, 350 s/mm^2^ were first replaced with outlier-free image volumes generated by a “pre-run” of EDDY correction, where EDDY function was performed in the same way as described earlier, but with the “–repol” option and without slice-to-volume correction.

The T_1_-weighted data were first corrected for the gradient nonlinearity induced image distortion in the same way as was performed for the diffusion data. For de-identification, facial features were obscured in each image volume^64^. Spatially varying intensity biases were then corrected using the unified segmentation routine^65^ implementation in the Statistical Parametric Mapping software (SPM) with a full-width at half-maximum of 30 mm and a sampling distance of 2 mm^66^. Finally, cerebral cortical surface reconstruction and volumetric segmentation were performed using the “recon-all” function of the FreeSurfer software^67–69^ (stable version v6.0.0). The volumetric segmentation results (i.e., aparc + aseg.mgz) were resampled to the diffusion image space using the affine transformation derived using the boundary-based registration^70^ implemented in FreeSurfer’s “bbregister” function and the averaged b = 0 image with the nearest neighbor interpolation.

### Diffusion model fitting

As an initial demonstration of the variety of analyses that can be performed with this comprehensive dataset, several diffusion models were fitted for estimating fiber orientations and microstructural metrics. These models are widely adopted in clinical and neuroscientific studies with publicly available software and open-source codes. Many other models such as diffusion basis spectrum imaging (DBSI)^71,72^ and soma and neurite density imaging (SANDI)^41^ can be also fitted, potentially on data from different diffusion times to explore the diffusion time dependence. The dataset also provides a unique opportunity for advancing the integration of diffusion MRI-based microstructural imaging and tractography, e.g., microstructure-informed tractography^25,73–75^ or tractography-informed microstructural imaging^76^. Despite recent interest in multi-modal data acquisition, i.e., combining diffusion MRI data with other MRI contrasts such as relaxometry and myelin-sensitive imaging measures, the focus of the current dataset is on the strengths of high-gradient diffusion MRI for microstructural imaging and thus does not include data from other contrasts, which has been explored in other complementary studies^14^.

Specifically, fiber orientation estimation was performed using six different methods. Diffusion tensor imaging^77,78^ model fitting was performed using the “dtifit” function from FSL on the b = 800 and 1500 s/mm^2^ data from the Δ = 19 ms acquisition. The multi-shell multi-tissue constrained spherical deconvolution^79–81^ (MSMT-CSD) was performed using the “dwi2response” function with the “msmt_5tt” option and the “dwi2fod” function with the “msmt_csd” option from the MRtrix3 software on the b = 800, 1500, 2400, 3450 s/mm^2^ data from the Δ = 19 ms acquisition. The segmentation of five tissue types was performed on the T_1_-weighted data using MRtrix3’s “5ttgen” function with the “fsl” option. All and stick (three sticks) model^82,83^ was fitted using FSL’s “BEDPOSTX” function on the b = 800, 1500, 2400, 3450 s/mm^2^ data from the Δ = 19 ms acquisition. The constant solid angle q-ball imaging^84^ (CSA-QBI) and the generalized q-space imaging^85^ (GQI) reconstruction was performed using the “CsaOdfModel” and “GeneralizedQSamplingModel” models from the Diffusion Imaging In Python (DIPY) software^86^, respectively, on the b = 6000 s/mm^2^ data from the Δ = 19 ms acquisition. Generalized diffusion spectrum imaging^27^ (GDSI) reconstruction was performed on the b = 50, 350, 800, 1500, 2400, 3450, 4750, 6000 s/mm^2^ data from the Δ = 19 ms acquisition using in-house Matlab codes. Default parameters of fitting/reconstruction functions were employed for all models.

In addition to DTI, multi-shell data from Δ = 19 ms acquisition (b = 800, 1500, 2400, 3450 s/mm^2^) were used to derive the microstructural metrics pertaining to five exemplary diffusion models as described below. Diffusion kurtosis imaging^87,88^ (DKI) and white matter tract integrity^89^ (WMTI) imaging model fitting was performed using the “dki_fit” function from the DESIGNER software. DKI and WMTI metrics were then derived using DESIGNER’s “dki_parameters” and “wmti_parameters” functions, respectively. The neurite orientation dispersion and density imaging (NODDI)^90^ metrics were derived using the NODDI Matlab toolbox. Microscopic DTI^91^ (MicroDTI) and Multi-compartment microscopic diffusion imaging^92^ (MCMicro) metrics were derived using the “fitmicrodt” and “fitmcmicro” functions from the SMT software.

## Data Records

The preprocessed diffusion MRI data and the T_1_-weighted MRI data of 26 healthy participants and the preprocessed rescan diffusion MRI data and the T_1_-weighted MRI data from seven of the 26 participants are publicly available through the figshare repository^93^. For participants sub_001 to sub _007, the rescan data are available (i.e., sub_001_rescan to sub _007_rescan). For participants sub_001 to sub_014, the real-valued diffusion data are available. The data of each participant are organized into a folder with 2 (participants with only magnitude-valued data) or 3 (participants with both magnitude-valued and real-valued data) folders as described below.

Folder 1: anat: T_1_-weighted MRI data corrected for gradient nonlinearity induced image distortion as well as a copy also corrected for spatially varying intensity bias. The estimated spatially varying intensity bias, brain mask, and volumetric segmentation results from FreeSurfer reconstruction (i.e., aparc + aseg.mgz) are also provided.

Folder 2: dwi: The pre-processed magnitude-valued diffusion MRI data along with the corresponding b-value and b-vector files. The brain mask, FreeSurfer volumetric segmentation results (i.e., aparc + aseg.mgz) resampled to the diffusion image space are also provided.

Folder 3: diff_real: The pre-processed real-valued diffusion MRI data, along with the corresponding b-value and b-vector files. The brain mask, FreeSurfer volumetric segmentation results (i.e., aparc + aseg.mgz) resampled to the diffusion image space are also provided.

In addition, the FreeSurfer reconstruction results and the image co-registration results (i.e., affine transformation matrices) between the diffusion and T_1_-weighted image space derived using boundary-based registration of each participant are provided in the “derivatives” folder.

## Technical Validation

### Assessment of diffusion data quality

**Fig. 2:**
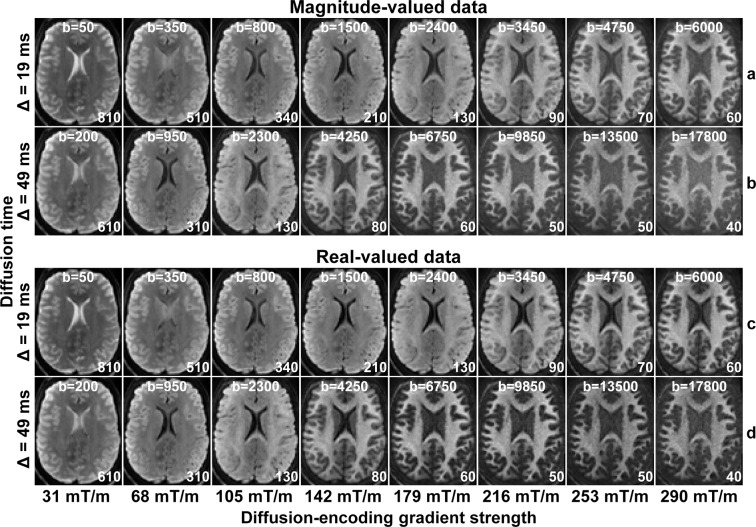
Spherical mean diffusion-weighted images. Spherical mean diffusion-weighted images for magnitude-valued (rows a, b) and real-valued (rows c, d) diffusion MRI data for each diffusion time (Δ) and diffusion-encoding gradient strength of a representative healthy participant. The images are windowed individually for improved visualization, between 0 and a higher image intensity listed at the bottom right of each sub-figure.

The magnitude-valued and real-valued spherical mean DWIs from each diffusion time and gradient strength of a representative participant are shown in Fig. 2. There were no significant ghosting or reconstruction artifacts by visual inspection. The image data for all participants were visually inspected for quality control. The difference between the magnitude-valued and real-valued spherical mean DWIs becomes visually discernible at b = 3450 s/mm^2^ and becomes increasingly prominent with increasing b-value. Specifically, the elevated Rician noise floor in the magnitude-valued data is removed in the real-valued data, which is characterized by a Gaussian noise distribution. The temporal signal-to-noise ratio (tSNR) quantified using the 50 interspersed b = 0 images, defined as the mean of time-series values over the standard deviation of time-series values at each voxel, was estimated in each participant and shown to be consistent across participants. The mean tSNR within the white matter was estimated to be 22.2 in a representative participant shown in Fig. 2. The group mean and group standard deviation of white matter-averaged tSNR was estimated to be 23.1 ± 2.46.

**Fig. 3:**
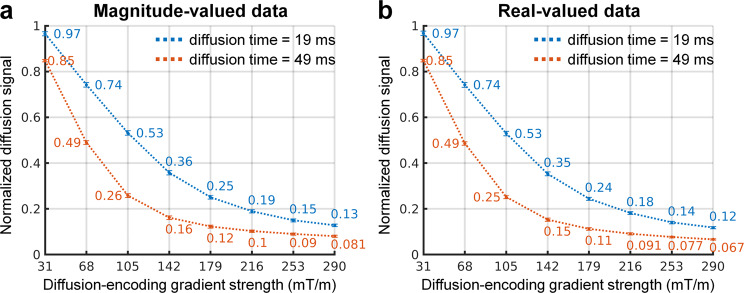
Normalized diffusion signal attenuation. The group mean (numbers below and above curves) and group standard deviation of the white matter-averaged normalized diffusion signal for each diffusion time and diffusion-encoding gradient strength.

The group mean and group standard deviation of the white matter-averaged normalized diffusion signal for each diffusion time and gradient strength are shown in Fig. 3. The normalized diffusion signal smoothly decays with increasing diffusion-encoding gradient strength. The real-valued signal is slightly lower than the magnitude-valued signal, and the difference is greater for longer diffusion times and higher diffusion-encoding strengths.

### Assessment of participant head motion

**Fig. 4:**
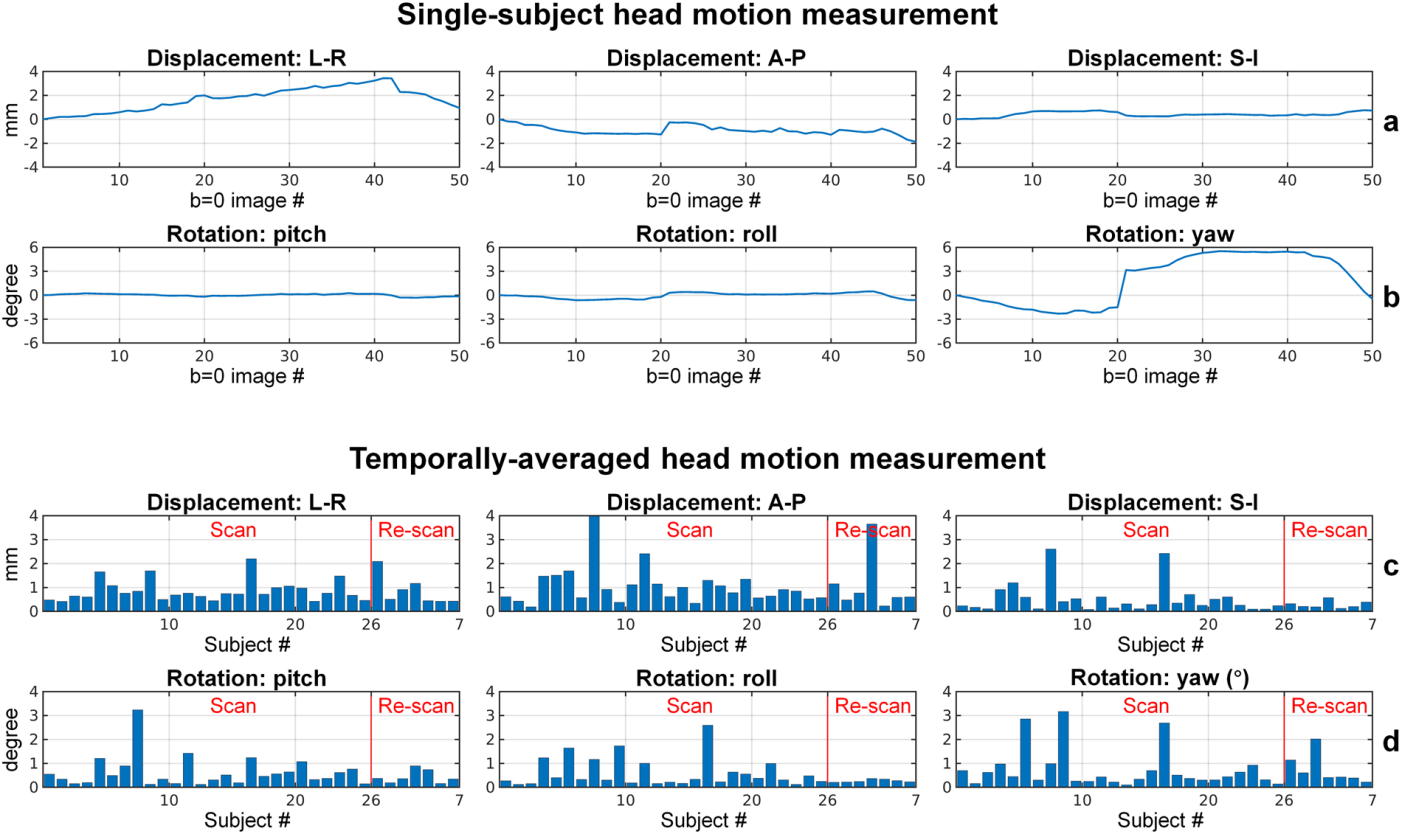
Participant head motion measurement. Head motion measurement estimated from the rigid-body co-registration between the 49 interspersed b = 0 image volumes and the first b = 0 image volume of a representative participant, quantified in the translation along the left-right (L-R), anterior-posterior (A-P), superior and inferior (S-I) direction (row a), as well as the rotation around the L-R axis (pitch), A-P axis (roll) and S-I axis (yaw) (row b). The temporally averaged absolute values of head motion parameters are used to reflect the overall head motion of each participant (rows c, d).

Participant head motion during the scan was evaluated, as shown in Fig. 4. The motion parameters estimated by FSL’s EDDY function from the rigid-body co-registration between the interspersed b = 0 image volumes and the first b = 0 image volume include the translation along the left-right (L-R), anterior-posterior (A-P), superior and inferior (S-I) direction (Fig. 4, row a), as well as the rotation around the L-R axis (pitch), A-P axis (roll) and S-I axis (yaw) (Fig. 4, row b). The temporally averaged absolute values of the motion measurements, which reflect the overall participant motion during the approximately one-hour scan, were relatively low. For most participants, the mean absolute translations along the L-R and A-P direction were confined within 1 mm, and the translation along S-I direction was within 0.5 mm. The group mean and group standard deviation for the translations along L-R, A-P and S-I directions were 0.86 ± 0.47 mm, 1.06 ± 0.93 mm and 0.49 ± 0.58 mm, respectively. For most participants, the mean absolute rotations were within 1 degree. The group mean and group standard deviation for the rotations around the L-R, A-P and S-I axis were 0.59 ± 0.59 degree, 0.54 ± 0.56 degree and 0.74 ± 0.79 degree.

The small to moderate motion and image distortions were successfully corrected by the pre-processing pipeline, quantified by the image correlation between the interspersed b = 0 image volumes and the first b = 0 image volume. For all participants, the image correlation between any b = 0 image volume and the first b = 0 image volume was higher than 0.95. The group mean and group standard deviation of the temporally averaged b = 0 image correlation was 0.84 ± 0.11 and 0.99 ± 0.0051 before and after the pre-processing.

### Assessment of diffusion modelling outcomes

**Fig. 5:**
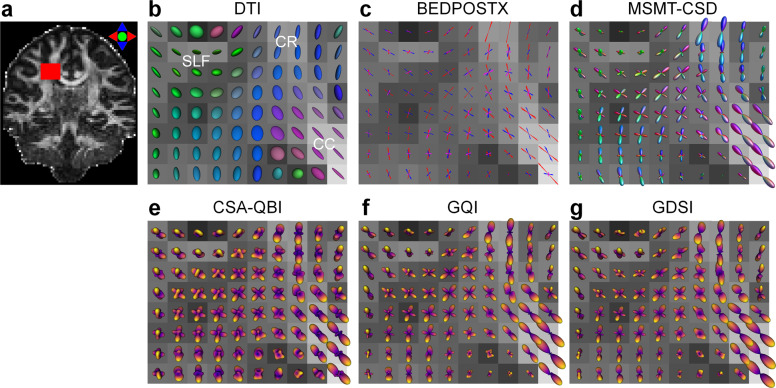
Fiber orientation estimation results. The diffusion tensor derived using the diffusion tensor imaging (DTI) (**b**), fiber orientation samples estimated from the BEDPOSTX method (10 randomly selected samples, stick length proportional to the fiber volume fraction) (**c**), and the orientation distribution functions (ODFs) reconstructed using the multi-shell multi-tissue constrained spherical deconvolution (MSMT-CSD) (**d**), the constant solid angle q-ball imaging (CSA-QBI) (**e**), the generalized q-space imaging (GQI) (**f**), and the generalized diffusion spectrum imaging (GDSI) (**g**) in the right centrum semiovale region (red rectangle in a) of a representative participant. The centrum semiovale region contains intersection of the corpus callosum (CC), the corona radiata (CR), and the superior longitudinal fasciculus (SLF). The diffusion tensors and MSMT-CSD ODFs are color coded based on orientation (red: left-right, green: anterior-posterior, blue: superior-inferior). The red, blue and magenta vectors from BEDPOSTX represent the primary, secondary and tertiary diffusion orientations, respectively. All reconstruction results are displayed on top of the DTI fraction anisotropy map (windowed between 0 and 1).

The quality of the diffusion MRI data is also assessed by evaluating the diffusion modelling outcomes using different methods. In terms of fiber orientation estimation, Fig. 5 shows the fiber orientation distributions within the right centrum semiovale of a representative participant estimated using six methods, including DTI, MSMT-CSD, BEDPOSTX, CSA-QBI, GQI and GDSI. These methods have different data requirements for the estimation, which are all satisfied by this comprehensive dataset. Except for DTI, which can only recover a single orientation, all other methods successfully recovered the fiber crossing at the intersecting region of three major fiber bundles: the corpus callosum, the corona radiata, and the superior longitudinal fasciculus. The mapped crossing fiber orientations from different methods were visually similar.

**Fig. 6:**
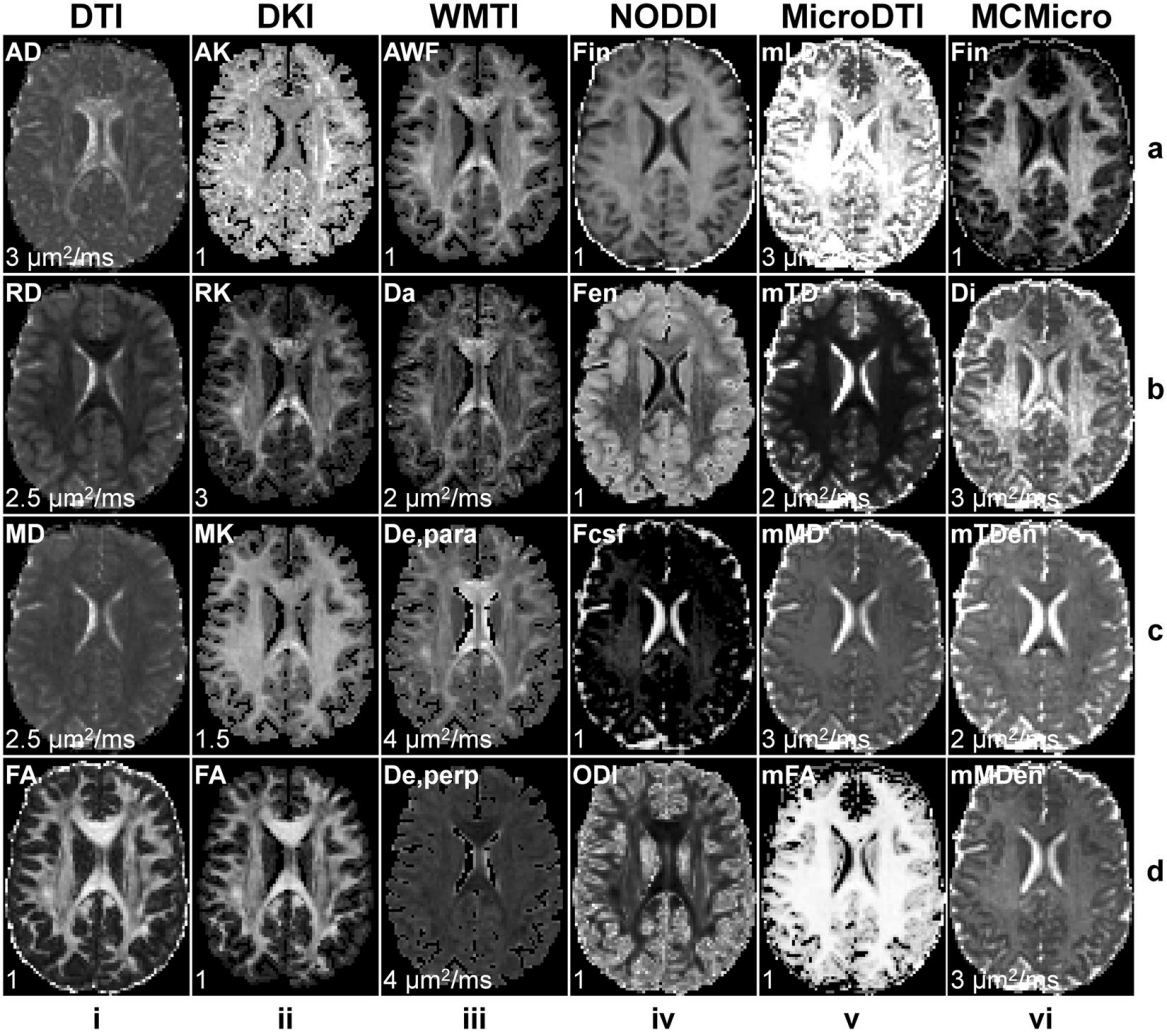
Microstructural modelling results. Maps of metrics estimated using various microstructural models of a representative participant. For diffusion tensor imaging (DTI) (column i), maps of axial diffusivity (AD), radial diffusivity (RD), mean diffusivity (MD) and fractional anisotropy (FA) are displayed. For diffusion kurtosis imaging (DKI), maps of axial kurtosis (AK), radial kurtosis (RK), mean kurtosis (MK), and FA from the tensor component are displayed. For white matter tract integrity (WMTI) imaging (column iii), maps of axonal water fraction (AWF), intra-axonal diffusivity (Da), axial extra-axonal diffusivity (De, para), and radial extra-axonal diffusivity (De, perp) are displayed. For neurite orientation dispersion and density imaging (NODDI) (column iv), maps of intra-neurite volume fraction (Fin), extra-neurite volume fraction (Fen), cerebrospinal fluid volume fraction (Fcsf), and orientation dispersion index (ODI) are displayed. For microscopic diffusion tensor imaging (MicroDTI) (column v), maps of microscopic longitudinal diffusivity (mLD), microscopic transverse diffusivity (mTD), microscopic mean diffusivity (mMD), and microscopic FA (mFA) are displayed. For multi-compartment microscopic diffusion (MCMicro) imaging (column vi), maps of intra-neurite volume fraction (Fin), intrinsic diffusivity (Di), microscopic extra-neurite transverse diffusivity (mTDen), microscopic extra-neurite mean diffusivity (mMDen) are displayed. The maps are windowed individually for improved visualization, between 0 and a higher intensity listed at the bottom left of each sub-figure.

Figure 6 shows maps of different microstructural metrics derived from six distinct diffusion modelling methods, including DTI, DKI, WMTI, NODDI, MicroDTI, and MCMicro. Specifically, maps of axial diffusivity (AD), radial diffusivity (RD), mean diffusivity (MD) and fractional anisotropy (FA) are displayed for DTI. Maps of axial kurtosis (AK), radial kurtosis (RK), mean kurtosis (MK), and FA from the tensor component are displayed for DKI. Maps of axonal water fraction (AWF), intra-axonal diffusivity (Da), axial extra-axonal diffusivity (De, para), and radial extra-axonal diffusivity (De, perp) are displayed for WMTI imaging. Maps of intra-neurite volume fraction (Fin), extra-neurite volume fraction (Fen), cerebrospinal fluid volume fraction (Fcsf), and orientation dispersion index (ODI) are displayed for NODDI. Maps of microscopic longitudinal diffusivity (mLD), microscopic transverse diffusivity (mTD), microscopic mean diffusivity (mMD), and microscopic FA (mFA) are displayed for MicroDTI. Maps of intra-neurite volume fraction (Fin), intrinsic diffusivity (Di), microscopic extra-neurite transverse diffusivity (mTDen), microscopic extra-neurite mean diffusivity (mMDen) are displayed for MCMicro imaging. These metrics are consistent with the expected results of respective models.

## Data Availability

The Matlab codes for gradient nonlinearity correction have been modified into Python codes, which are publicly available (https://github.com/ksubramz/gradunwarp). The FMRIB Software Library used in the diffusion data pre-processing and DTI and BEDPOSTX model fitting is publicly available (https://fsl.fmrib.ox.ac.uk/fsl/fslwiki/FslInstallation). The FreeSurfer software is publicly available (https://surfer.nmr.mgh.harvard.edu/fswiki/DownloadAndInstall). The software used for face masking is publicly available (https://nrg.wustl.edu/software/face-masking). The SPM software for image intensity bias correction is publicly available (https://www.fil.ion.ucl.ac.uk/spm). The MRtrix3 software for the constrained spherical deconvolution is publicly available (https://www.mrtrix.org). The DIPY software used for the CSA-QBI and GQI reconstruction is publicly available (https://www.dipy.org). The codes for the GDSI reconstruction are publicly available (https://github.com/qiyuantian/GDSI). The DESIGNER software for the DKI and WMTI model fitting is publicly available (https://github.com/NYU-DiffusionMRI/DESIGNER). The NODDI Matlab toolbox is publicly available (http://mig.cs.ucl.ac.uk/index.php?n=Tutorial.NODDImatlab). The SMT software for the MicroDTI and MCMicro model fitting are publicly available (https://github.com/ekaden/smt).
